# Protein deglycase DJ‐1 deficiency induces phenotypic switching in vascular smooth muscle cells and exacerbates atherosclerotic plaque instability

**DOI:** 10.1111/jcmm.16311

**Published:** 2021-01-27

**Authors:** Zhao‐yang Wang, Jie Cheng, Bin Liu, Fei Xie, Chang‐ling Li, Wen Qiao, Qing‐hua Lu, Ying Wang, Ming‐xiang Zhang

**Affiliations:** ^1^ The Key Laboratory of Cardiovascular Remodeling and Function Research Chinese Ministry of Education and Chinese Ministry of Public Health Department of Cardiology Qilu Hospital of Shandong University Jinan China; ^2^ Department of Cardiology The Second Hospital of Shandong University Jinan China

**Keywords:** atherosclerotic plaque, phenotype switching, plaque stability, protein deglycase DJ‐1

## Abstract

Protein deglycase DJ‐1 (DJ‐1) is a multifunctional protein involved in various biological processes. However, it is unclear whether DJ‐1 influences atherosclerosis development and plaque stability. Accordingly, we evaluated the influence of DJ‐1 deletion on the progression of atherosclerosis and elucidate the underlying mechanisms. We examine the expression of DJ‐1 in atherosclerotic plaques of human and mouse models which showed that DJ‐1 expression was significantly decreased in human plaques compared with that in healthy vessels. Consistent with this, the DJ‐1 levels were persistently reduced in atherosclerotic lesions of ApoE^−/−^ mice with the increasing time fed by western diet. Furthermore, exposure of vascular smooth muscle cells (VSMCs) to oxidized low‐density lipoprotein down‐regulated DJ‐1 in vitro. The canonical markers of plaque stability and VSMC phenotypes were evaluated in vivo and in vitro. DJ‐1 deficiency in Apoe^−/−^ mice promoted the progression of atherosclerosis and exaggerated plaque instability. Moreover, isolated VSMCs from Apoe^−/−^DJ‐1^−/−^ mice showed lower expression of contractile markers (α‐smooth muscle actin and calponin) and higher expression of synthetic indicators (osteopontin, vimentin and tropoelastin) and Kruppel‐like factor 4 (KLF4) by comparison with Apoe^−/−^DJ‐1^+/+^ mice. Furthermore, genetic inhibition of KLF4 counteracted the adverse effects of DJ‐1 deletion. Therefore, our results showed that DJ‐1 deletion caused phenotype switching of VSMCs and exacerbated atherosclerotic plaque instability in a KLF4‐dependent manner.

## 1 INTRODUCTION

Atherosclerosis, the primary cause of cardiovascular disease and the top cause of death globally, is a complex disease initiated by the accumulation of lipids in the subendothelial layer of the arterial wall.^1^, ^2^, ^3^, ^4^ The most important clinical consequence of atherosclerosis is acute rupture or erosion of unstable plaques in which vascular smooth muscle cells (VSMCs) constitute the primary cell type; this finding has been verified by lineage‐tracing experiments and single‐cell sequencing.^5^, ^6^, ^7^, ^8^, ^9^, ^10^, ^11^ During this pathological process, VSMCs change from a contractile phenotype to a de‐differentiated phenotype (also called a synthetic phenotype), marked by reductions in various unique contractile proteins (eg α‐smooth muscle actin [SMA], calponin1, smooth muscle 22α and smooth muscle myosin heavy chain) and higher rates of migration and proliferation.^8^, ^12^, ^13^, ^14^, ^15^, ^16^, ^17^ Many studies have evaluated the mechanisms and factors involved in VSMC phenotype switching.

Protein deglycase DJ‐1 (DJ‐1), a 189‐amino acid protein (also known as PARK7), was first identified as an original oncogene accountable for the autosomal recessive early‐onset form of Parkinson's disease.^18^, ^19^, ^20^, ^21^ As a multifunctional protein, DJ‐1 is ubiquitously distributed and play essential roles in various biological process.^22^, ^23^ Although DJ‐1 is positively associated with tumours, several studies have found that DJ‐1 protects cells from different harmful stimuli. For example, mutations in DJ‐1 result in neurodegeneration, leading to an early‐onset familial form of Parkinson's disease.^20^, ^24^, ^25^ Moreover, overexpression of DJ‐1 defends against oxidative stress‐induced damage, whereas models of the absence of DJ‐1 are more susceptible to cerebral ischaemia, neuronal cell death, hypertension and obesity owing to increased oxidative stress.^24^, ^26^, ^27^, ^28^, ^29^ Using proteomics analysis, Won et al showed that DJ‐1/PARK7 responds rapidly to oxidative stress in VSMCs.^30^, ^31^ Subsequently, they demonstrated that DJ‐1 inhibits neointimal hyperplasia and maintains vasorelaxation by participating in the synthesis of endothelial nitric oxide synthase.^32^ Altogether, these findings denoted that DJ‐1 has vital functions in the vascular system. However, the association between DJ‐1 and atherosclerosis and the direct mechanisms through which DJ‐1 affects VSMC phenotype switching remains unclear.

Accordingly, in this study, we evaluated the impact of DJ‐1 deletion on atherosclerotic plaque stability and clarified the potential mechanisms.

## 2 MATERIALS AND METHODS

### 2.1 Materials

The following primary antibodies were used in this study: anti‐β‐actin (cat. no. 13E5; Cell Signaling Technology, Danvers, MA, USA), anti‐DJ‐1 (cat. no. ab76088; Abcam, Cambridge, UK), anti‐αSMA (cat. no. ab14106; Abcam), anti‐calponin (cat. no. ab46794; Abcam), anti‐osteopontin (cat. no. ab8448; Abcam), anti‐tropoelastin (cat. no. ab21600; Abcam), anti‐vimentin (cat. no. D21H3; Cell Signaling Technology), anti‐CD68 (cat. no. ab201340; Abcam), Anti‐ p44 / 42 MAPK, phospho (Erk1 / 2) Duet – PhosphoPlus (cat. no. 8201; Cell Signaling Technology) and anti‐Kruppel‐like factor 4 (KLF4; cat. no. ab214666 from Abcam; cat. no. D1F2 from Cell Signaling Technology). The following secondary antibodies were used in this study: rabbit/mouse Alexa Fluor 488 (cat. nos. A‐11034 and A‐11001; Thermo Fisher Scientific, Waltham, MA, USA) and rabbit/goat Alexa Fluor 594 (cat. nos. A‐11005 and A27016; Thermo Fisher Scientific). ERK inhibitor (SCH772984; Catalog No.S7101) was purchased from selleckchem (USA). Oil red O (cat. no. O0625) was purchased from Sigma‐Aldrich (Shanghai, China). Lipofectamine RNAiMAX Reagent was purchased from Thermo Fisher Scientific. The western diet (cat. no. TP28640) was purchased from Trophic Animal Feed High‐Tech Co., Ltd. (China), and oxidized low‐density lipoprotein (ox‐LDL; cat. no. YB‐0010) was purchased from Yiyuan Biotech (Guangzhou, China).

### 2.2 Animal experiments

All animal experiments were completed regarding the rules approved by the Institutional Committee for the Use and Care of Laboratory Animals of Qilu Hospital, Shandong University. ApoE^–/–^ mice were purchased from Beijing Viewsolid Biotech Co; Ltd. All mice were male and of the C57BL/6J background. For genotype detection in ApoE^–/–^ mice, the following primers were employed: oIMR180 (5′‐GCCTAGCCGAGGGAGAGCCG‐3′), oIMR181 (5′‐TGTGACTTGGGAGCTCTGCAGC‐3′) and oIMR182 (5′‐GCCGCCCCGACTGCATCT‐3′). DJ‐1^–/–^ mice were generated at the Model Animal Research Center of Nanjing University, and their genotypes were determined using the following primers: 5′‐GATAGCTTTCCGGGACACAC‐3′; 5′‐TCCATCAGCTCCTCCACCTCT‐3′; 5′‐TAAGTTGGGTAACGCCAGGGT‐3′). ApoE^–/–^DJ‐1^–/–^ mice were generated by crossbreeding DJ‐1^–/–^ mice with ApoE^–/–^ mice in our laboratory and were identified using the above two primers. The mice were maintained in a fully controlled and specific‐pathogen‐free animal room (12/12‐hour light/dark cycle, 22°C).

### 2.3 Tissue harvesting and preparation

Mice were anaesthetized at different time‐points by intraperitoneal injection of pentobarbital natrium (40 mg/kg bodyweight). We peeled adherent adipose tissue off the whole aortas and aortic roots. The aortas and aortic roots were then immediately fixed in 4% paraformaldehyde for subsequent experiments. Next, aortic root samples were embedded using OCT (Fisher, Tustin, CA, USA) or paraffin and serially sectioned at 8 μm thickness.

### 2.4 Human tissue harvesting

Human coronary arteries were obtained from Qilu Hospital of Shandong University based on the protocols for human studies approved by the institutional ethical committee.

### 2.5 Oil Red O staining

Entire arteries were longitudinally opened, incubated in Oil Red O solution for 2‐4 hours and then incubated in 70% ethyl alcohol until the arteries turned red. After being rinsed with 60% isopropanol, frozen sections were stained with freshly prepared Oil Red O working solution for 15 minutes. The staining area of Oil Red O was measured as a percentage of the total section area. The average size of lipid droplets was calculated using ImagePro Plus software (Media Cybernetics, Rockville, MD, USA) from five views per mouse.

### 2.6 Immunohistochemistry

Tissue samples were incubated with primary antibodies at a dilution of 1:200. After that, they were incubated with a goat anti‐mouse/‐rabbit peroxidase‐labelled antibody (ZSGB‐BIO) as a secondary antibody for 30 minutes. Histopathological slide analysis was performed with ImagePro Plus software.

### 2.7 Immunofluorescence staining of sections

The immunofluorescence staining was examined using 7‐mm frozen sections of the aortic roots. The sections were blocked with 5% normal donkey serum (Dako) at room temperature for 1 hour, followed by the incubation of primary antibodies (1:200) at 4°C overnight. After 1 hour incubation with the second antibody at room temperature, the sections were then washed three times with phosphate‐buffered saline (PBS), and the nucleus was co‐stained with 4′,6‐diamidino‐2‐phenylindole (DAPI).

### 2.8 Cell culture and Small interfering RNA (siRNA) transfection

Primary mouse VSMCs were isolated from aortae of 8‐week‐old mice (from the aortic roots to bifurcation of the renal arteries) as described.^33^ VSMCs were cultured in complete Dulbecco's modified Eagle's medium at 37°C in an atmosphere containing 5% CO_2_.

The transient transfection of siRNA into VSMCs was achieved using Lipofectamine RNAi MAX transfection reagent (Thermo Fisher Scientific) according to the manufacturer's instructions.

### 2.9 Lentivirus vectors for DJ‐1

Lentiviral vectors containing green fluorescence protein (GFP) were employed. Recombined pGC‐LV‐GV287‐GFP vector with full length of the mouse DJ‐1 (NCBI reference sequence ID, NM_020569) gene (LV‐DJ‐1) and pGC‐LV‐GV287‐GFP with a scrambled control sequence (LV‐NC) were constructed by Genechem Company (Genechem, Shanghai, China).

*Quantitative real‐time reverse transcription polymerase chain reaction (qRT‐PCR)* Total RNA was extracted using RNeasy Mini Kit (Qiagen, Valencia, CA, USA) and reverse‐transcribed into cDNA using the Prime Script RT reagent Kit (Takara, Dalian, China). Individual qRT‐PCR was carried out using specific primers, as described in the Supplement files.

### 2.10 Statistical analysis

Data are expressed as means and standard errors of the means. Statistical analysis was carried out through one‐way analysis followed by Tukey's tests with SPSS statistical software package (version 22.0; SPSS, Chicago, IL, USA). Results with *P* values of less than 0.05 were considered significant.

## 3 RESULTS

### 3.1 DJ‐1 expression was reduced in human atherosclerotic carotid arteries

**FIGURE 1:**
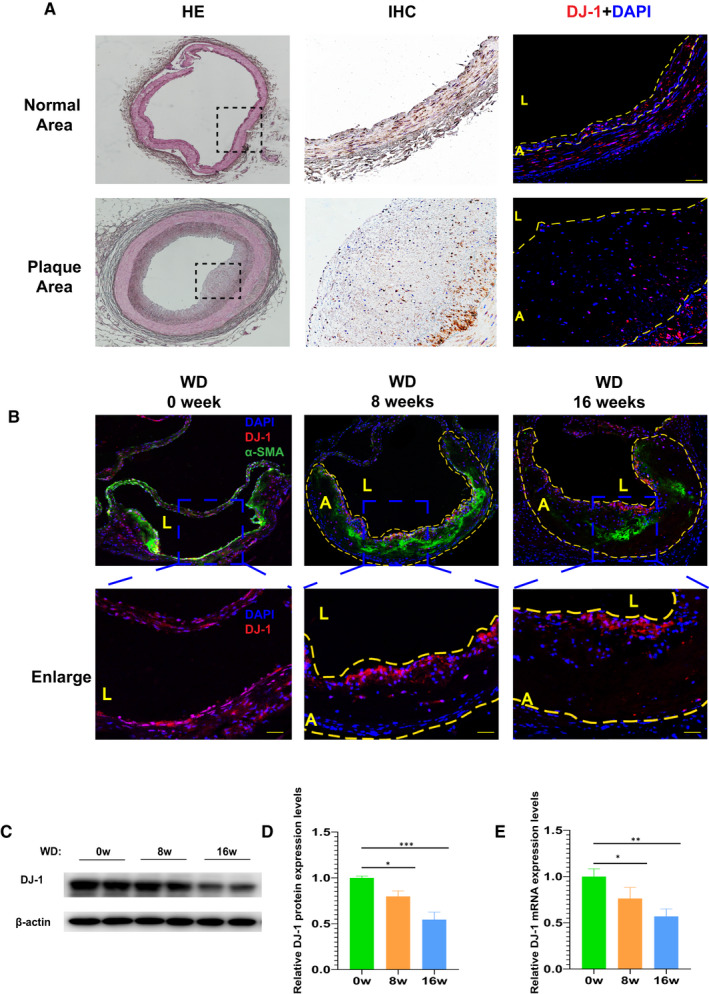
Expression of DJ‐1 was reduced during atherosclerosis. A, Immunofluorescence and immunochemistry staining of DJ‐1 in normal human arteries and atherosclerotic lesion of carotid arteries (n = 6). B, Immunostaining images of aortic root lesions from ApoE^–/–^ mice fed a western diet for the indicated times stained for DJ‐1 (red), SM‐α‐actin (green) and with DAPI (blue) (scale bar, 50 µm; n = 5). Dashed line denotes the plaque necrotic core region. L indicates the lumen; A, atheroma. Blue box indicates the enlarged part of the aortic roots which showed in the lower panel (scale bar, 50 µm; n = 5). (C,D) Western blot analysis of DJ‐1 protein expression in the aortas of ApoE^–/–^ mice fed a western diet for the indicated times. E, Quantitative qRT‐PCR analysis of *DJ‐1* mRNA levels in ApoE^−/−^ mice (n = 7; *P < 0.05; **P < 0.01; ***P < 0.001)

To prove whether DJ‐1 expression was involved in atherosclerosis, we detected the expression of DJ‐1 in healthy human vessels and carotid atherosclerotic plaques using immunochemistry and immunofluorescence staining. As shown in Figure 1A, the expression of DJ‐1 was apparently lower in atherosclerotic plaques, which suggested a crucial role of DJ‐1 in the progress of atherosclerotic plaques.

### 3.2 Time‐dependent decrease of DJ‐1 in ApoE^−/−^ mice fed by western diet

Next, we assessed DJ‐1 expression in ApoE^−/−^ mice with a western diet for different times. As illustrated in Figure 1B, immunofluorescence staining showed that western diet feeding resulted in a significant reduction in the expression of DJ‐1 and α‐SMA, a marker of smooth muscle cells, in the atherosclerotic area. Additionally, after 8 weeks of high‐fat diet, the protein levels of DJ‐1 were decreased by 25% compared with mice fed a chow diet. Furthermore, a 50% reduction of DJ‐1 protein expression was found after consumption of western diet for16 weeks (Figure 1C,D). Similar to the protein expression, the *DJ‐1* mRNA levels showed a continuous reduction with the time of western diet prolonged (Figure 1E). The above results indicated the DJ‐1 expression decreased with the progression of atherosclerosis.

### 3.3 DJ‐1 was down‐regulated by ox‐LDL in VSMCs in vitro

**FIGURE 2:**
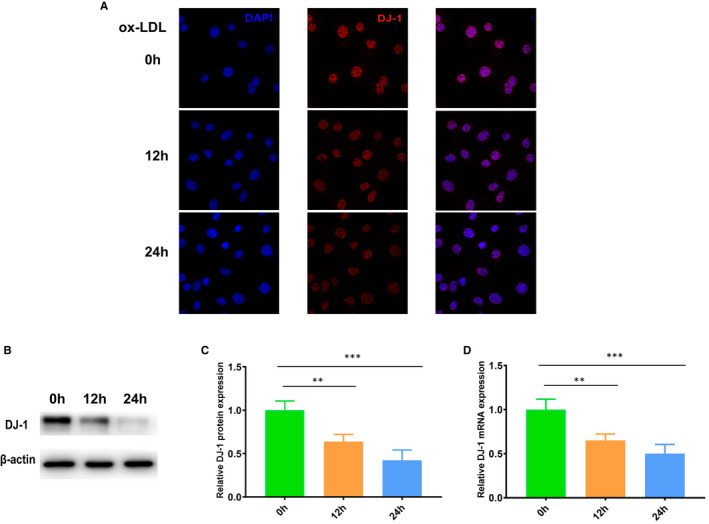
DJ‐1 was down‐regulated by oxidized low‐density lipoprotein (ox‐LDL) in smooth muscle cells in vitro. A, Immunostaining of DJ‐1 expression in VSMCs treated with ox‐LDL (100 µg/mL) for the indicated times. (B,C) Western blot analysis of DJ‐1 protein expression in VSMCs treated with ox‐LDL for the indicated times. D, Quantitative qRT‐PCR analysis of *DJ‐1* mRNA levels in VSMCs (n = 6; *P < 0.05; **P < 0.01; ***P < 0.001)

VSMCs are one of the vital constitutes in the development of atherosclerosis.^6^, ^7^, ^34^ To assess the influence of atherosclerosis on DJ‐1 expression in VSMCs, VSMCs were cultured with the atherosclerotic stimulus ox‐LDL. Immunofluorescence imaging showed that DJ‐1 expression was continuously down‐regulated over time following stimulation with ox‐LDL (Figure 2A). Consistent with this, as shown in Figure 2B,C, DJ‐1 protein levels were decreased to 64% after 12 hours of ox‐LDL treatment in VSMCs and were further reduced to 42% after 24 hours of ox‐LDL treatment. Similar findings were observed using qRT‐PCR (Figure 2D). These findings further demonstrated that DJ‐1 expression in VSMCs was closely related to the development of atherosclerosis.

### 3.4 DJ‐1 deletion accelerated the progression of atherosclerosis

**FIGURE 3:**
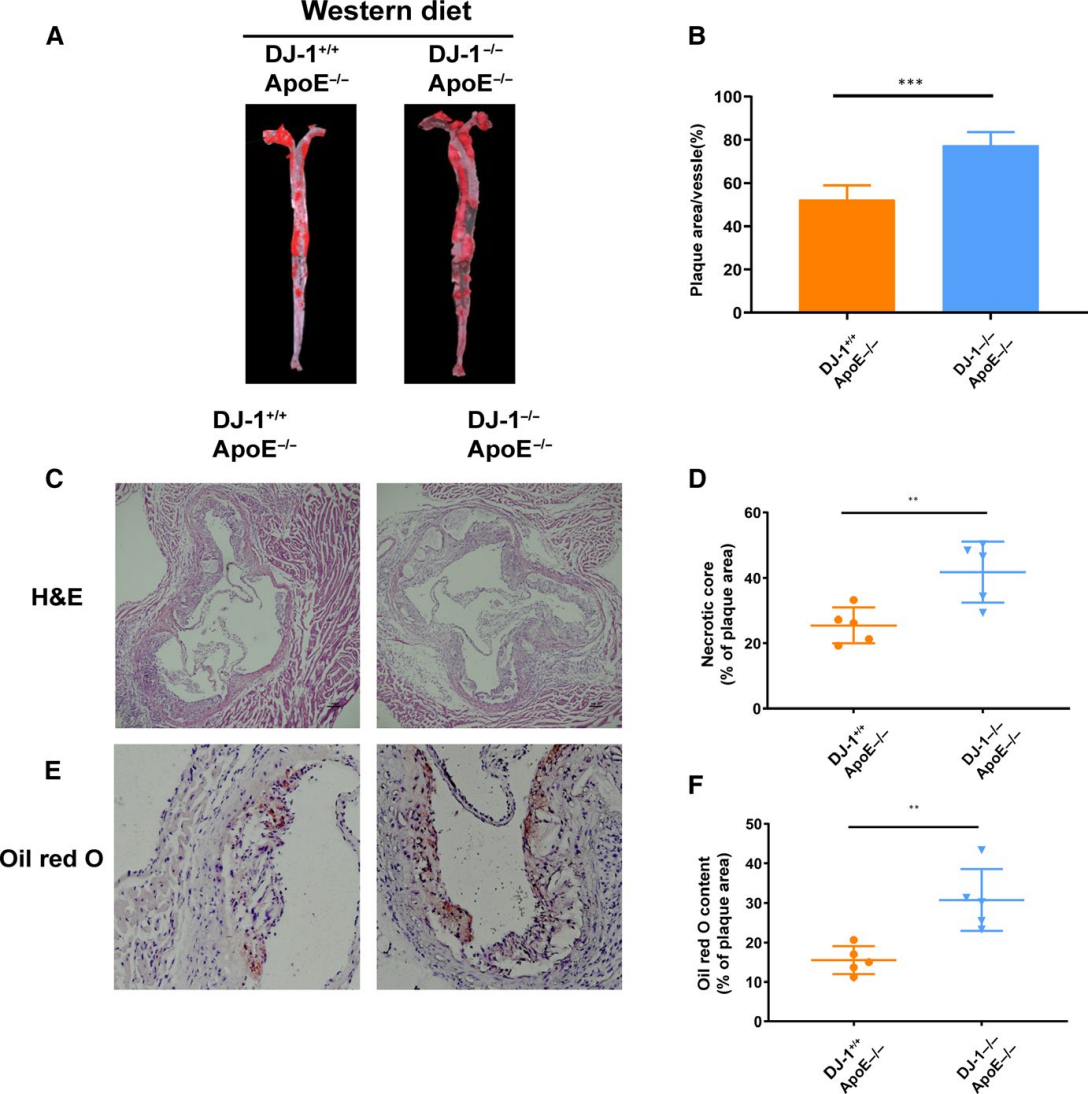
DJ‐1 deletion accelerated the progression of atherosclerosis. ApoE^–/–^DJ‐1^–/–^ mice and ApoE^–/–^DJ‐1^+/+^ controls were fed a western diet for 16 weeks. (A,B) Representative images of Oil Red O staining of the whole aorta. The lesion area was quantified as the percentage of the total surface area of the aorta (n = 5 per genotype). (C,D) Representative haematoxylin‐ and eosin‐stained images and quantification of necrotic core areas in the aortic root. The necrotic core area was quantified as a percentage of the plaque area (n = 5 per genotype). (E,F) Oil Red O staining of aortic root lesions (scale bar, 200 µm; n = 5 per genotype; **P < 0.01; ***P < 0.001)

Based on the above results, we used ApoE^−/−^DJ‐1^−/−^ mice fed a western diet to explore the influences of DJ‐1 on atherosclerotic plaque development. By performing lesion analysis in en face aortas, we observed that ApoE^−/−^DJ‐1^−/−^ mice showed an increase in the atherosclerotic burden compared with littermate controls (ApoE^−/−^DJ‐1^+/+^; Figure 3A,B). Besides, we use the Lv‐DJ‐1 to overexpressed DJ‐1 in ApoE^−/−^ mouse and found that the ApoE^−/−^ mouse with DJ‐1 overexpression showed a significant decrease in atherosclerotic burden (Figure SD1A). Moreover, haematoxylin and eosin staining showed that the area of the necrotic core in ApoE^−/−^DJ‐1^−/−^ mice was much larger than that in ApoE^−/−^DJ‐1^+/+^ mice (Figure 3C,D). Oil Red O staining of aortic roots also indicated that atherosclerosis was exacerbated in ApoE^−/−^DJ‐1^−/−^ mice (Figure 3E,F). In conclusion, these results demonstrated that DJ‐1 plays a protective role in atherosclerosis and deficiency of DJ‐1 could evidently accelerate the progression of atherosclerosis.

### 3.5 DJ‐1 deletion exacerbated atherosclerotic plaque instability

**FIGURE 4:**
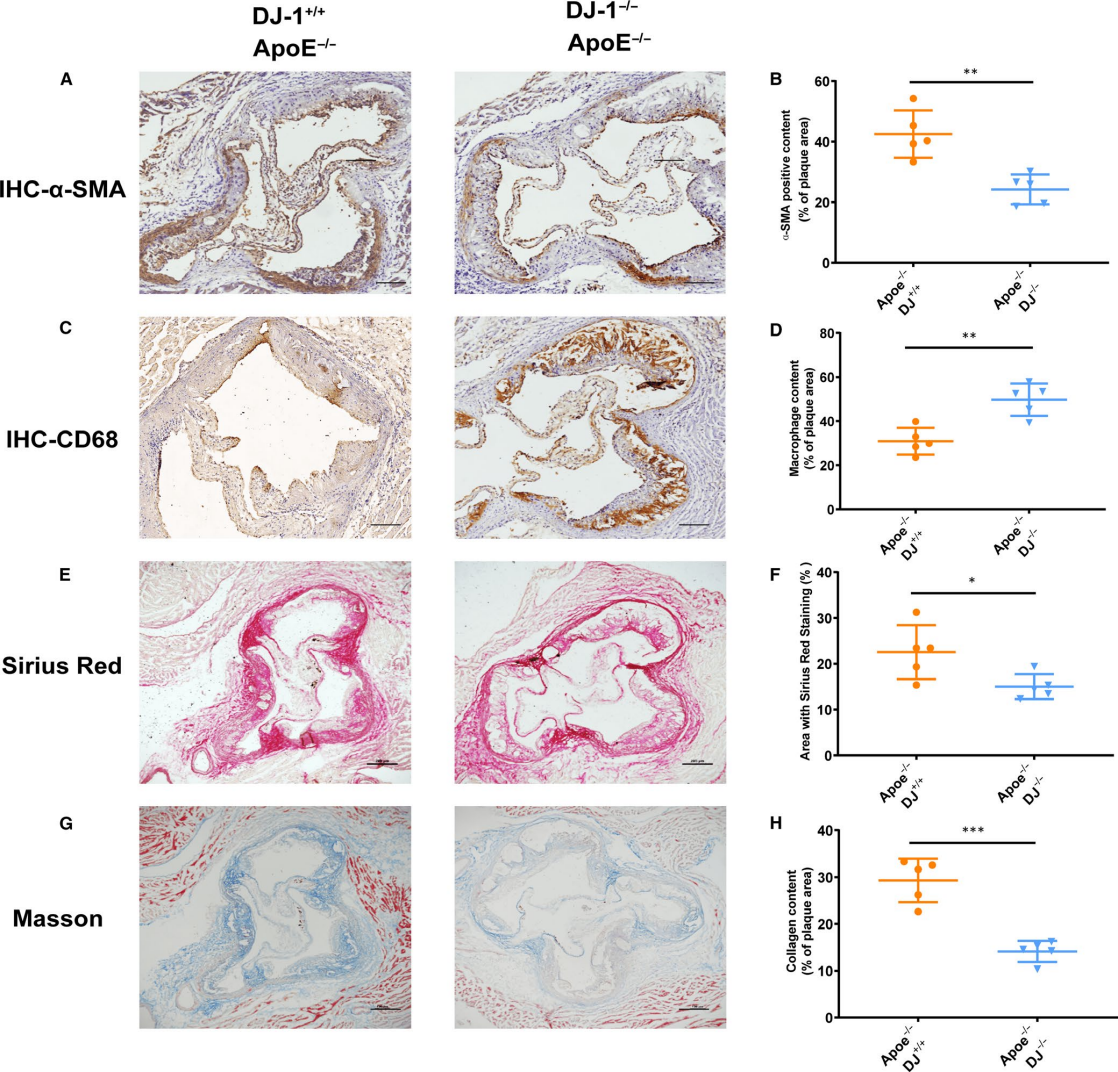
DJ‐1 deletion exacerbated atherosclerotic plaque instability. (A,B) Representative α‐SMA immunohistochemistry staining and quantification of aortic root sections from all groups. Scale bars = 200μm. (C,D) Representative images and quantification of plaque macrophage content in aortic root sections based on CD68 immunohistochemistry. (E,F) Representative images from aortic roots of western diet‐fed ApoE^–/–^DJ‐1^–/–^ mice and ApoE^–/–^DJ‐1^+/+^ mice stained with Sirius Red and quantification for the fibrous cap area (red staining). (G,H) Representative images and quantification of plaque collagen content for sections of aortic roots using Masson trichrome staining (n = 5‐6 in each group; *P < 0.01; **P < 0.01; ***P < 0.001; scale bar = 200 µm)

To further elucidate the role of DJ‐1 in plaque stability, we use the Sirius red, Masson, CD68 and α‐SMA staining.^35^, ^36^ As shown in Figure 3E,F, the Oil Red O staining area, as an indicator of atherosclerotic burden, was significantly increased in ApoE^−/−^DJ‐1^−/−^ mice compared with that in Apoe^−/−^ mice fed a western diet. In addition, the proportion of smooth muscle cells significantly decreased in ApoE^−/−^DJ‐1^−/−^ mice (Figure 4A,B). Furthermore, macrophage content was increased by almost twofold in Apoe^−/−^DJ‐1^−/−^ mice compared with that in controls (Figure 4C,D). Moreover, the Sirius red and Masson staining revealed that the collagen area was dramatically decreased in ApoE^−/−^DJ‐1^−/−^ mice, which are widely used indicators of plaque stability (Figure 4E–H). And as Figure S1B illustrated, VSMCs deficient in DJ‐1 expressed more MMP2, MMP9 and less Collagen I, Collagen III protein which could explain the decreased collagen in ApoE^−/−^DJ‐1^−/−^ mice. All the above results considered, the existence of DJ‐1 could efficiently prevent the progression of atherosclerosis and increased the stability of atherosclerotic plaques.

### 3.6 DJ‐1 deficiency induced VSMC phenotypic switching

**FIGURE 5:**
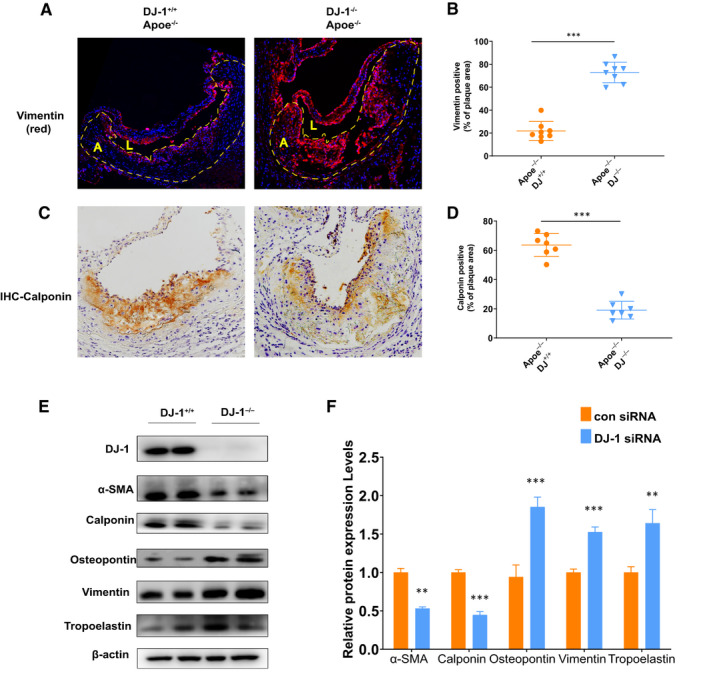
DJ‐1 deficiency induced VSMC phenotypic switching. A, Representative images of immunofluorescence staining of vimentin in aortic root sections of ApoE^–/–^DJ‐1^–/–^ mice and ApoE^–/–^DJ‐1^+/+^ mice. Dashed line denotes the plaque necrotic core region. L indicates the lumen; A, atheroma. (DAPI = blue staining of nuclei; scale bar = 200 µm). B, Quantification of vimentin‐positive content in total plaques of aortic root sections. C, Representative images of immunochemistry staining for calponin in aortic root sections of ApoE^–/–^DJ‐1^–/–^ mice and ApoE^–/–^DJ‐1^+/+^ mice. Scale bar = 50 µm. D, Quantification of calponin content in total plaques from aortic root sections of ApoE^–/–^DJ‐1^–/–^ mice and ApoE^–/–^DJ‐1^+/+^ mice. (E,F) Western blot analysis of contractile (α‐SMA and calponin) and synthetic proteins (vimentin, osteopontin and tropoelastin) in VSMCs isolated from ApoE^–/–^DJ‐1^–/–^ mice and ApoE^–/–^DJ‐1^+/+^ mice (n = 5‐6 in each group; **P < 0.01; ***P < 0.001 versus DJ‐1^+/+^ controls)

Aberrant proliferation and phenotype switching of VSMCs contribute to the progression of atherosclerosis.^6^, ^8^, ^16^, ^37^ As demonstrated by immunohistochemical analysis (Figure 4A,B), α‐SMA, a typical marker of contractile VSMCs, was significantly decreased in the fibrous caps and total plaques in ApoE^−/−^DJ‐1^−/−^ mice compared with that in controls. Therefore, we next evaluated whether DJ‐1 expression affected the phenotypic switching of VSMCs by examining the expression of critical markers during VSMC phenotypic switching. As illustrated in Figure 5A,B, ApoE^−/−^DJ‐1^−/−^ mice showed a higher expression of vimentin compared with the controls. In contrast, calponin, a marker of contractile VSMCs, was decreased threefold in ApoE^−/−^DJ‐1^−/−^ mice compared with that in controls (Figure 5C,D).

Furthermore, we isolated smooth muscle cells from the aortas of ApoE^−/−^DJ‐1^−/−^ and ApoE^−/−^ mice to elucidate the mechanisms. As shown in Figure 5E,F, the expression levels of α‐SMA and calponin dramatically declined in DJ‐1^−/−^ VSMCs compared with those in control VSMCs, whereas protein levels of osteopontin, vimentin and tropoelastin, three well‐characterized markers of synthetic VSMCs, were markedly increased in DJ‐1^−/−^ VSMCs (Figure 5E,F). And VSMCs overexpressing DJ‐1 by Lv‐DJ‐1 showed the opposite trend which favours the contractile phenotype smooth muscle cells (Figure S2A). Collectively, our results indicated that DJ‐1 deletion elicited the phenotypic alteration of VSMCs in vitro and in vivo.

### 3.7 DJ‐1 deficiency induced VSMC phenotype switching via the ERK/KLF4 pathway

KLF4, a known transcription factor of KLF family, is a critical negative regulator of VSMC contractile proteins.^38^, ^39^ Therefore, we reasoned that DJ‐1 deletion may increase KLF4 expression to promote VSMC phenotypic switching and plaque instability.

**FIGURE 6:**
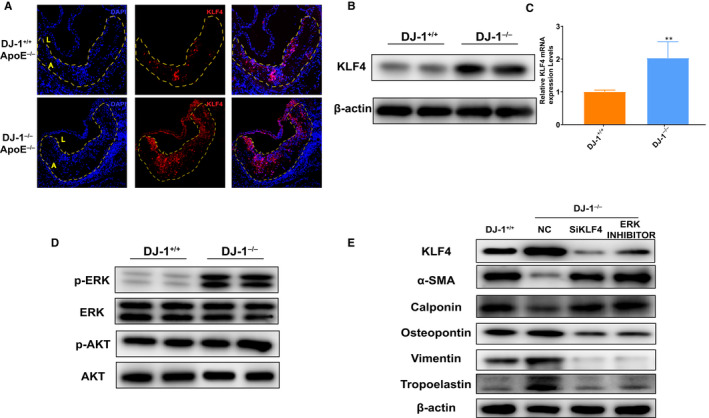
DJ‐1 deficiency promotes VSMCs phenotypic switch via increasing KLF4 expression in an ERK‐dependent way. A, Immunofluorescence staining of KLF4 expression in aortic root sections of ApoE^–/–^DJ‐1^–/–^ mice and ApoE^–/–^DJ‐1^+/+^ mice. Dashed line denotes the plaque necrotic core region. L indicates the lumen; A, atheroma. (DAPI = blue staining of nuclei; scale bar = 50 µm) (B,C) Western blot and qRT‐PCR analysis of KLF4 protein expression in VSMCs from DJ‐1^–/–^ and DJ‐1^+/+^ mice (n = 5; **, *P* < 0.01 versus DJ‐1^+/+^). D, Western blot analysis protein expression in VSMCs from DJ‐1^–/–^ and DJ‐1^+/+^ mice (n = 5). E, Western blot analysis of the expression of contractile (α‐SMA and calponin) and synthetic proteins (vimentin, osteopontin and tropoelastin) in DJ‐1^+/+^ VSMCs and DJ‐1^–/–^ VSMCs treated with KLF4 siRNA and SCH772984 (n = 5)

To validate the hypothesis, we measured KLF4 expression in plaques from aortic roots and VSMCs. As shown in Figure 6A, the KLF4‐positive area in aortic roots of DJ‐1‐deletion groups markedly increased compared with the controls. Additionally, similar results were acquired using Western blotting and qRT‐PCR. Therefore, the above findings demonstrated that DJ‐1 deficiency increased KLF4 expression.

**FIGURE 7:**
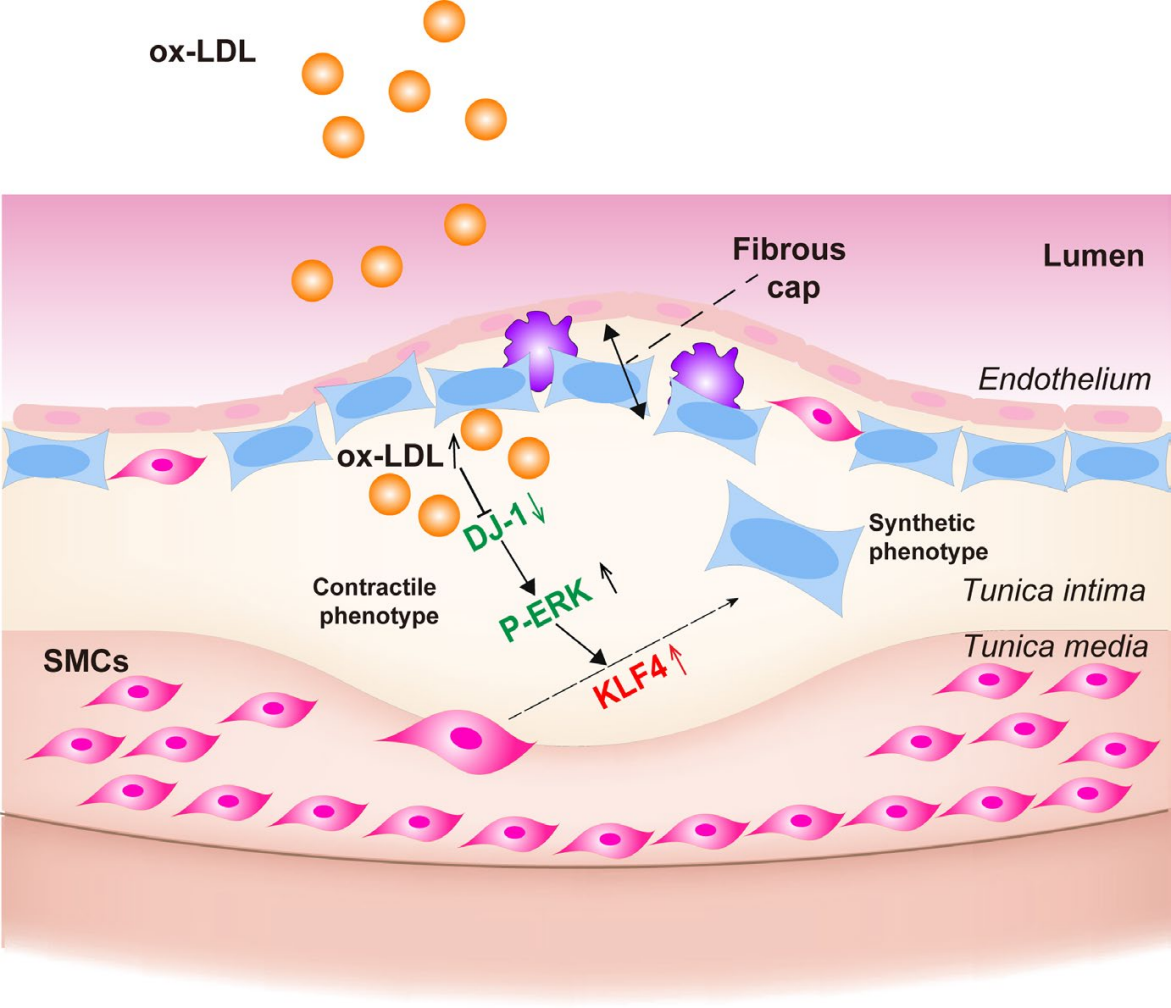
DJ‐1 signalling in vascular smooth muscle cells. DJ‐1 modulates VSMCs phenotypic switch DJ‐1/ERK/KLF4 signalling pathway

To further assess whether DJ‐1 deletion induced VSMC phenotype transition through the KLF4 pathway, primary aortic VSMCs from control or DJ‐1^−/−^ mice were transfected with KLF4 siRNA to down‐regulate KLF4 expression. As expected silencing of KLF4 in DJ‐1^−/−^ VSMCs prevented the reduction in protein levels of α‐SMA and calponin and blocked the up‐regulation of synthetic markers (Figure S2B). To explore the mechanism of how DJ‐1 influences KLF4 expression, we focused on the common upstream kinases in the KLF4 signalling pathway, such as ERK1/2, AKT.^40^, ^41^, ^42^, ^43^ To confirm the involvement of ERK1/2 and AKT in DJ‐1 effects on KLF4 expression in VSMCs, we firstly analysed the expression of ERK1/2, AKT and their phosphorylated form among which significant increased EKR1/2 were found in DJ‐1 deficiency VSMCs (Figure 6D). We further use ERK1/2 inhibitor to verify the hypothesis and found ERK1/2 inhibitor could effectively ameliorate the DJ‐1 deficiency caused up‐regulated KLF4 and inhibit the VSMCs phenotype transition towards synthetic type (Figure 6E). Therefore, we conclude that DJ‐1 prevented the synthetic alteration of VSMCs via KLF4 in an ERK1/2‐dependent way. (Figure 7).

## 4 DISCUSSION

In this study, we found that DJ‐1 deficiency accelerated the development of atherosclerosis and exacerbated the instability of atherosclerotic plaques. Additionally, our results showed that DJ‐1 regulated the VSMC phenotypic switch towards synthetic type negatively. Mechanistically, down‐regulation of DJ‐1 controlled the phenotypic change in VSMCs via the KLF4 pathway. Consistent with these findings, genetic inhibition of KLF4 eliminated the phenotypic switching induced by DJ‐1 deletion. Importantly, overexpression of DJ‐1 strongly ameliorated the down‐regulation of contractile proteins in isolated primary VSMCs treated with atherosclerotic stimuli. Taken together, these results suggested that DJ‐1 played an essential role in protecting against VSMC phenotype switching and atherosclerosis progression.

DJ‐1 has been shown to be closely related to the vascular system and to protect cells from oxidative stress.^23^, ^24^, ^26^, ^32^ However, few studies have evaluated the relationship between DJ‐1 and atherosclerosis. Here, we found that DJ‐1 expression was decreased following the exposure to pro‐atherosclerosis stimuli in vitro and along the course of atherosclerosis. Additionally, in ApoE^−/−^DJ‐1^−/−^ mice, genetic inactivation of DJ‐1 enhanced the features of the late period of atherosclerosis.

Vascular smooth muscles, as a hallmark of atherosclerosis, experience remarkable phenotypic switching in vitro, as has been verified using lineage‐tracing experiments in vivo.^5^, ^44^, ^45^ Moreover, VSMC migration also makes an important attribution to the cumulation of VSMCs in advanced plaques, where the cells form neointima after vascular injury.^7^, ^46^ Recently, Won et al reported that DJ‐1 has a protective role in neointimal hyperplasia by inhibiting VSMC growth.^30^ In this study, we showed that DJ‐1 deficiency promoted phenotypic switching of VSMCs in vivo and in vitro. However, some findings from previous studies were inconsistent with our current work. For example, Won demonstrated that DJ‐1 expression was up‐regulated in mouse aortic smooth muscle strips treated with 300 mmol/L H_2_O_2_ for 30 minutes and in mouse neointimal plaque tissues after 3 weeks of carotid artery ligation. The disparities may be related to the different time periods of stimulus in vitro and differences in time‐points in the animal model of human disease.

KLF4 plays a crucial role in VSMC differentiation, and a growing number of papers have identified KLF4 could inhibit VSMC switching towards synthetic phenotype via binding directly to TGFβ control element within the SM a‐actin promoter and to the G/C‐repressive element within the SM22a and SM‐MHC promoters and inhibits the cooperative interaction of paired CArG elements located in the 50‐region of virtually all SMC marker genes.^47^, ^48^, ^49^, ^50^ Moreover, Liu showed that KLF4 is rapidly induced in VSMCs in vivo in response to vascular injury.^51^ Therefore, based on the previous study, we assumed KLF4 as a target of DJ‐1 in modulating the phenotype transition and use si‐KLF4 to validate the effect of KLF4 on VSMCs phenotype transition. In this study, we found that genetic inhibition of DJ‐1 significantly increased KLF4 expression. And SCH772984 could efficiently ameliorate the DJ‐1 deficiency caused up‐regulation of KLF4 and switch to synthetic phenotype. In summary, these results demonstrated that DJ‐1 deficiency induced VSMC phenotype switching via the ERK/KLF4 pathway.

Furthermore, we also demonstrated that DJ‐1 deficiency significantly enhances the vulnerability of atherosclerotic plaque area in ApoE^−/−^DJ‐1^−/−^ mice; especially, the collagen content decreased dramatically in ApoE^−/−^DJ‐1^−/−^ mice. As evidence suggests MMP‐2 and MMP‐9 are the main proteases involved in atherogenesis and collagen types I and III are the main constitution of collagen in atherosclerotic plaque. ^52^, ^53^, ^54^, ^55^, ^56^ We found VSMCs deficient in DJ‐1 express more MMP‐2 and MMP9 whereas Collagen I and Collagen III decreased significantly using isolated VSMCs. These results showed that deficiency of DJ‐1 enhances the instability of plaque by influencing the matrix collagen and metalloproteinases. In conclusion, our results demonstrated a novel role of DJ‐1 in inhibiting the progression of atherosclerosis and unveiled a new theory underlying the control of phenotypic switching of VSMCs via ERK/KLF4. (Figure 7) These findings highlight the protecting role of DJ‐1 in atherosclerosis and provide a potential therapeutic target for reducing atherosclerosis and enhancing plaque stability.

## CONFLICT OF INTEREST

The authors confirm that there are no conflicts of interest.

## AUTHOR CONTRIBUTIONS

**Zhao‐yang Wang:** Conceptualization (lead); Data curation (lead); Formal analysis (lead); Investigation (lead); Methodology (lead); Resources (lead); Software (lead); Writing‐review & editing (equal). **Jie Cheng:** Data curation (equal); Methodology (equal); Supervision (equal); Visualization (equal). **Bin Liu:** Investigation (equal); Resources (equal); Visualization (equal). **Fei Xie:** Data curation (equal); Resources (equal); Software (equal). **Changling Li:** Methodology (equal); Software (equal); Validation (equal); Visualization (equal). **Wen Qiao:** Data curation (equal); Formal analysis (equal); Investigation (equal); Resources (equal); Visualization (equal). **Qing‐hua Lu:** Conceptualization (equal); Project administration (equal); Writing‐review & editing (supporting). **Ying Wang:** Conceptualization (lead); Formal analysis (lead); Methodology (equal); Project administration (lead); Software (equal); Supervision (lead); Validation (lead). **Ming‐xiang Zhang:** Conceptualization (lead); Funding acquisition (lead); Project administration (lead); Supervision (lead); Writing‐review & editing (lead).

## Supporting information

Fig S1

Fig S2

## Data Availability

The data that support the findings of this study are available from the corresponding author upon reasonable request.
