# Supplementary figures and images for: Protein deglycase DJ‐1 deficiency induces phenotypic switching in vascular smooth muscle cells and exacerbates atherosclerotic plaque instability
Source: J Cell Mol Med. 2021 Jan 27;25(6):2816–27. doi: 10.1111/jcmm.16311 (PMC7957272; doi:10.1111/jcmm.16311)

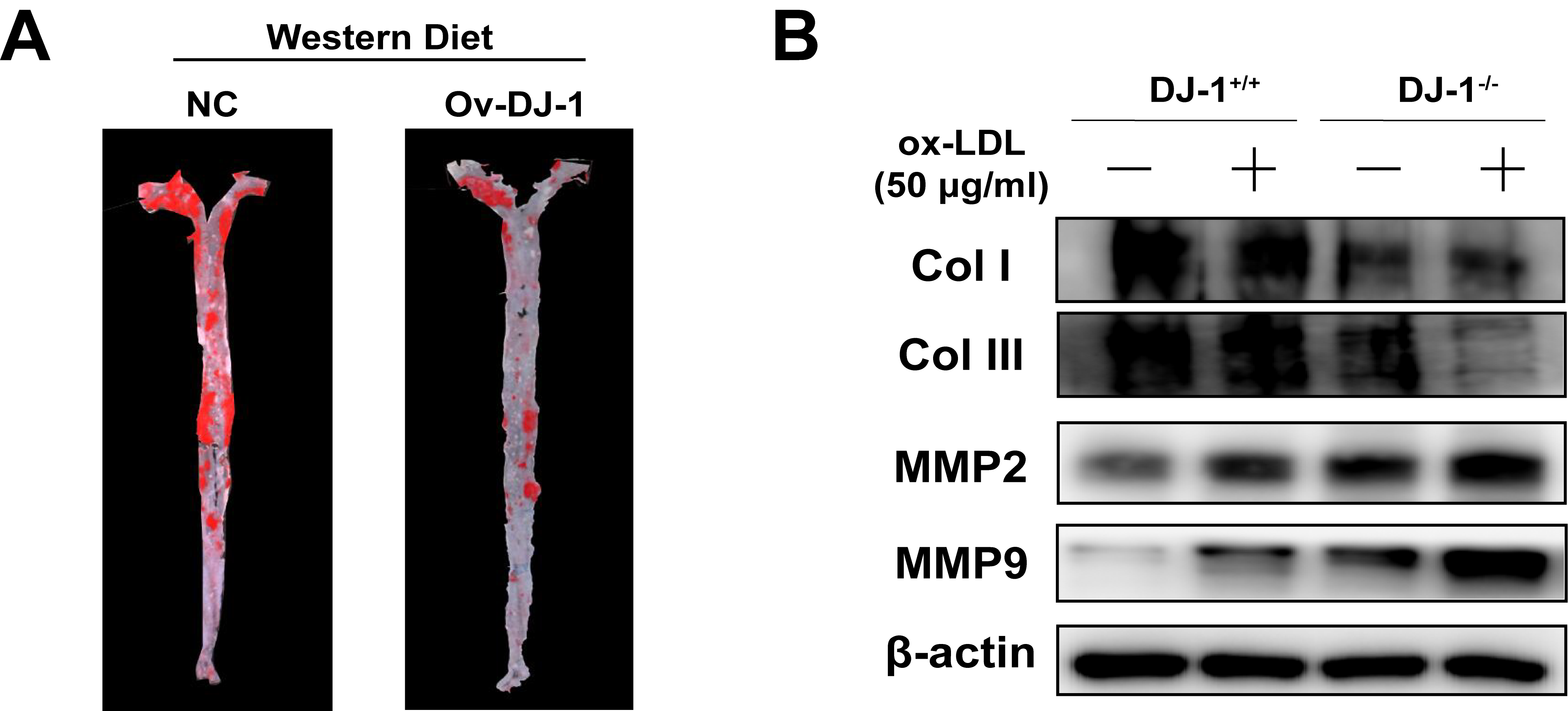

Supplement: Supplementary file 1 — Fig S1 [file JCMM-25-2816-s002.tif]

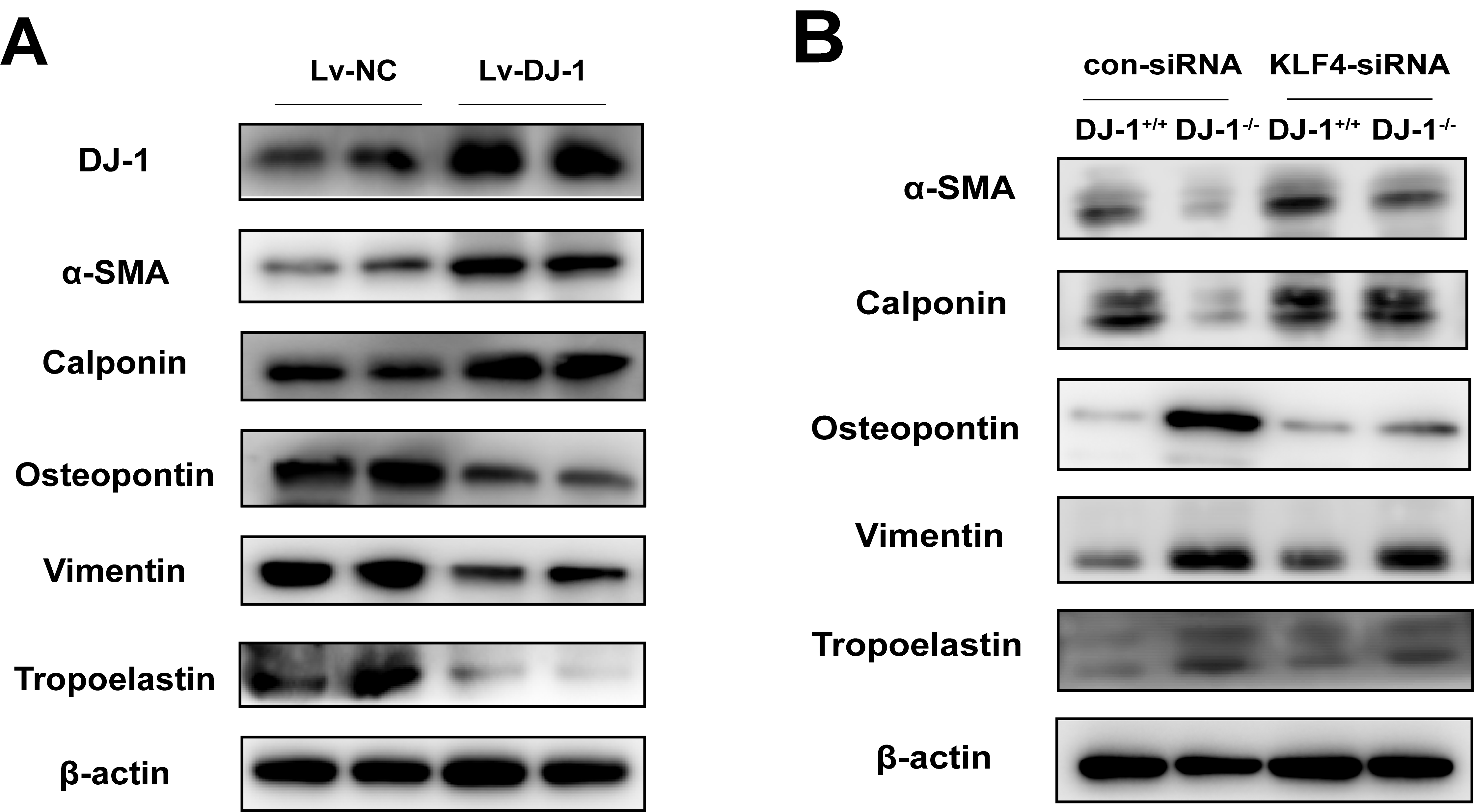

Supplement: Supplementary file 2 — Fig S2 [file JCMM-25-2816-s001.tif]
